# High-Efficiency Lossy Source Coding Based on Multi-Layer Perceptron Neural Network

**DOI:** 10.3390/e27101065

**Published:** 2025-10-14

**Authors:** Yuhang Wang, Weihua Chen, Linjing Song, Zhiping Xu, Dan Song, Lin Wang

**Affiliations:** 1 Navigation College, Jimei University, Xiamen 361021, China; 2 School of Ocean Information Engineering, Jimei University, Xiamen 361021, China; 3 Department of Information and Communication Engineering, Xiamen University, Xiamen 361005, China

**Keywords:** data compression, lossy source coding, P–LDPC codes, enhanced belief propagation, distortion–rate performance

## Abstract

With the rapid growth of data volume in sensor networks, lossy source coding systems achieve high–efficiency data compression with low distortion under limited transmission bandwidth. However, conventional compression algorithms rely on a two–stage framework with high computational complexity and frequently struggle to balance compression performance with generalization ability. To address these issues, an end–to–end lossy compression method is proposed in this paper. The approach integrates an enhanced belief propagation algorithm with a multi–layer perceptron neural network, aiming to introduce a novel joint optimization architecture described as “encoding–structured encoding–decoding”. In addition, a quantization module incorporating random perturbation and the straight–through estimator is designed to address the non–differentiability in the quantization process. Simulation results demonstrate that the proposed system significantly improves compression performance while offering superior generalization and reconstruction quality. Furthermore, the designed neural architecture is both simple and efficient, reducing system complexity and enhancing feasibility for practical deployment.

## 1. Introduction

With the rapid proliferation of applications such as environmental monitoring [1], industrial applications [2], and smart cities [3], sensor networks are generating data at an unprecedented scale, yet they operate under stringent constraints in edge computing capacity, communication bandwidth, and node energy budgets [4,5,6,7]. This fundamental mismatch between data volume and available resources intensifies the demand for high–throughput [8] and low–latency [9] data processing under limited computational capabilities. Furthermore, it necessitates the development of efficient transmission strategies that accommodate narrow bandwidth and multi–hop relaying [10], while avoiding congestion and excessive energy drain [11]. These challenges underscore the critical need for efficient data acquisition, in–situ processing, and low–overhead communication protocols in the development of scalable and sustainable sensor networks [12,13,14].

To address these constraints, data compression is often applied at sensor nodes to significantly reduce the bit rate within controllable distortion limits [15,16,17]. This approach effectively decreases link occupancy, end–to–end latency, and transmission energy consumption, while also alleviating back–end storage and processing burdens [18,19,20]. Among various compression strategies, the lossy source coding based on the rate–distortion trade–off offers a compelling framework for joint node and link level optimization by exchanging minor perceptual or statistical losses for subsantial compression gains [21].

Existing lossy source coding systems can be categorized into three main approaches. The first category relies on sparse–graph codes, which establish an operational duality between lossy compression and channel decoding [12]. Studies have shown that the low–density parity–check (LDPC) structures, combined with the belief propagation (BP) and its variants, can approach theoretical rate–distortion bounds for binary symmetric sources. The second category employs structured schemes based on protograph LDPC (P–LDPC) codes, which were initially validated on binary sources and later extended to noisy environments and continuous Gaussian sources [17]. These methods facilitate hardware reuse between source and channel coding blocks, thereby reducing overall system complexity. The third category integrates sparse graphs with neural networks in an end–to–end fashion, mapping sources into pseudo–codeword spaces for structured quantization and reconstruction [21]. However, the existing implementations are employed with shallow perceptrons, which limited both modeling capacity and generalization ability.

Hence, several critical challenges persist in current lossy source coding systems. The classical BP often requires numerous iterations during encoding and quantization, resulting in high computational complexity and latency that strain real–time processing capabilities and energy budgets [22]. Furthermore, the conventional two–stage “quantization–compression” pipelines are prone to error accumulation and bit–importance imbalance, leading to additional distortion and reduced coding efficiency [21]. Although the multi–level coding (MLC) can partially mitigate information inequality, it introduces higher architectural complexity and complicates rate allocation while still failing to achieve full equalization [23]. Moreover, the end–to–end learning–based approaches often rely on shallow neural architectures that lack explicit reconstruction mechanisms, thereby limiting their compression accuracy and generalization ability [24,25]. These limitations collectively exacerbate the constraints on delay, energy consumption, and link robustness in sensor network applications [26,27,28].

To address these issues, a novel lossy source coding system featuring an end–to–end “encoding–structured encoding–decoding” architecture is proposed. A multi–layer perceptron (MLP) is employed for nonlinear feature transformation and explicit reconstruction. Furthermore, an enhanced belief propagation (EBP) quantization module is incorporated with the random perturbation and a straight–through estimator (STE) to enable differentiable training. In the coding module, a tunable P–LDPC structure offers flexible support for a wide range of compression rates. In contrast to conventional two–stage pipelines and shallow learnable baselines, the proposed system achieves unidirectional end–to–end information flow, integrates an explicit trainable reconstruction, and delivers lower distortion with improved generalization, all while maintaining practical computational complexity and enhanced rate agility.

The remainder of this paper is structured as follows: Section 2 introduces the overall system framework by elaborating on the three core components of the system, including the encoding module, the structured encoding (quantization) module, and the decoding module. Section 3 discusses essential techniques for end–to–end training, including strategies for differentiable gradient propagation, and provides a comprehensive complexity analysis. Section 4 presents the experimental setup, comparative results, and evaluations of performance and generalization capability. Finally, Section 5 concludes the paper and suggests potential applications and future research directions.

## 2. System Model

**Figure 1:**
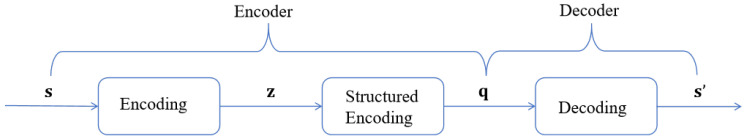
The proposed lossy source coding system based on a joint optimization architecture.

For clarity, the principal symbols and their meanings used in this section are summarized in Table 1. The proposed compression system integrates neural networks with P–LDPC code as shown in Figure 1. The system is composed of three main components, including the encoding, structured encoding, and decoding modules. Both the encoding and decoding modules are implemented using the MLP–based deep neural networks for nonlinear feature extraction and approximate reconstruction of the input data. The structured encoding module utilizes a quantization method based on the BP algorithm to discretize the continuous features output by the encoding module under the P–LDPC structural constraints to produce binary codewords.

In this system, the encoder comprises a cascaded structure formed by the encoding module and the quantization, while the decoder consists of a dedicated decoding module. The workflow begins with a Gaussian source sequence being transformed by a deep neural network within the encoding module to produce feature representations. These features are subsequently mapped into structured binary sequences via a BP–based quantization. Finally, the binary codes are approximately reconstructed by the neural network–based decoding module to recover the original signal. In contrast to conventional schemes, the proposed architecture eliminates feedback connections between the transformation and the quantization, adopting instead a unidirectional end–to–end information flow. This design enhances both model simplicity and inference efficiency.

The proposed system introduces key improvements over traditional frameworks. While existing algorithms perform well within LDPC structures, their direct application to Gaussian source compression often leads to high reconstruction distortion. To mitigate this, a deep MLP–based encoding module is integrated with a structured BP quantization, significantly enhancing representational capacity and reducing distortion. The architecture is fully end–to–end, allowing the encoder and decoder to adaptively reconstruct the original signal without prior assumptions by leveraging the strong approximation capabilities of deep networks. A differentiable approximation within the BP quantization is incorporated to overcome the non–differentiability of quantization for inhibiting the gradient propagation. In this case, the effective gradient flow of the encoder is optimized based on reconstruction loss. Furthermore, the system supports a wider range of compression rates by adjusting the P–LDPC matrix structure and parameters, which offers a versatile and efficient solution for lossy compression across diverse scenarios.

Referring to Figure 1, a memoryless Gaussian source sequence s of length *n* is first fed into the encoding module. The MLP–based encoding maps the input sequence into a continuous feature vector z of the same length. Subsequently, a BP–based structured encoding module performs a projection on z to generate a binary codeword q of length mn, which satisfies predefined P–LDPC constraints, where the length values satisfy m,n∈Z, and Z is a set of positive integers. After that, the quantified result q serves as the output of the encoder and is directly passed into the decoding module. The decoding module is also implemented by the MLP, aiming to reconstruct an approximate estimation $s^{\prime }$ of the original source s from q.

It is noteworthy that the proposed system employs a fully end–to–end and unidirectional information flow, eliminating any feedback connections from the quantization output. This design results in a more streamlined and efficient processing pipeline. The structure and functionality of each module are described in detail in the following sections.

### 2.1. Encoding Module

**Figure 2:**
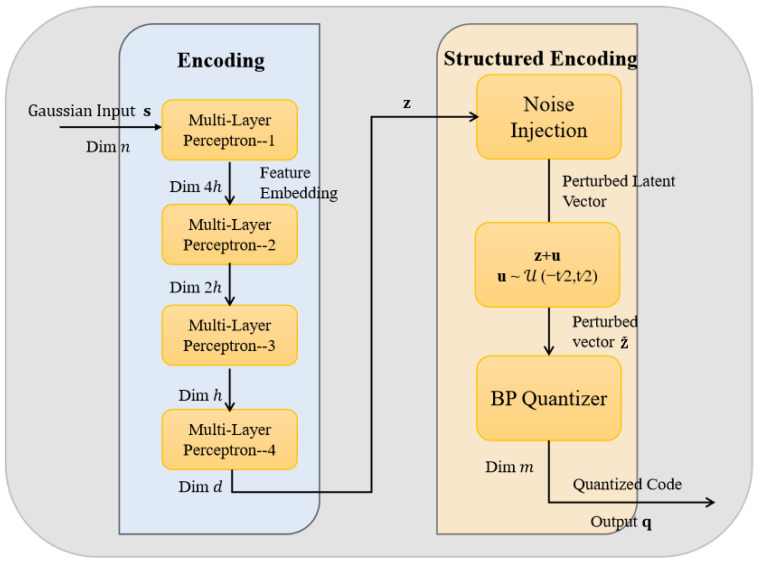
Detailed architecture of the proposed encoder integrating an MLP and a structured quantization module.

The encoder is composed of a cascaded encoding module and structured encoding module for realizing the transformation and quantization, respectively, as illustrated in Figure 2. For a memoryless Gaussian source sequence $s=\{s_{1},s_{2},\ldots ,s_{n}\}$ of length *n*, the feature extraction is first performed by the transformation module. This module employs the MLP to carry out deep nonlinear mapping of the input signal, resulting in a continuous feature representation as $z=\{z_{1},z_{2},\ldots ,z_{n}\}$. The continuous feature vector z is then fed into the structured quantization module, where z is quantified by the BP algorithm into a binary codeword $q=\{q_{1},q_{2},\ldots ,q_{n}\}$ that conforms to the structural constraints of the P–LDPC code. Here, the variables satisfy $s_{i},z_{i},q_{i}\in R$, i∈Z, and R is a set of real numbers.

The principle of the MLP structure is shown in Table 2. The parameter *n* denotes the length of the quantized binary codeword, and *d* represents the dimension of the reconstructed signal, where d∈Z. Different from traditional multi–stage architectures with feedback cascades, the proposed system employs a single end–to–end encoding pathway that operates without feedback from the quantization output or concatenation with the original input. Each signal undergoes transformation and quantization only once, significantly simplifying the encoder structure and enhancing both training and inference efficiency. Leveraging the powerful nonlinear transformation capability of the deep MLP, the transformation module produces highly expressive feature representations that effectively facilitate subsequent structured quantization and reconstruction.

Subsequently, the source sequence s is directly fed into the MLP neural network within the transformation module for the nonlinear transformation. The MLP employed in this work consists of multiple fully connected layers, as illustrated in Figure 2. Each linear layer is followed by batch normalization (BN) and a LeakyReLU function, while the output layer uses a Tanh function to constrain the output within a bounded range. Taking an input $s\in R^{p\times n}$ as an example, where *p* denotes the batch size and *n* is the dimensionality of the input data, and p∈Z. Hence, the transformation process of the MLP can be formulated as

(1) $$z=\mathrm{Tanh}\left(f_{L}\left(\cdots \left(f_{2}\left(f_{1}\left(s\right)\right)\right)\right)\right),$$

and the LeakyReLU function is defined as

(2) $$\mathrm{LeakyReLU}\left(x\right)=\left\{\begin{array}{cc} x, & \mathrm{if}\;\;x\geq 0,\\ \alpha x, & \mathrm{otherwise}, \end{array}\right.$$

where α is a small positive constant, for instance α=0.01, and each intermediate function $f_{i}\left(\cdot \right)$ is defined as

(3) $$f_{i}\left(x\right)=\mathrm{LeakyReLU}\left(\mathrm{BatchNorm}(xW_{i}+b_{i})\right),$$

where $W_{i}$ and $b_{i}$ denote the weight matrix and bias vector of the i–th fully connected layer, respectively.

Based on the MLP architecture, the transformation module effectively captures complex nonlinear features within the Gaussian source sequence and produces continuous feature representations that are well–suited for subsequent structured quantization. To further enhance network performance, the Xavier initialization method is adopted for parameter initialization. This strategy helps mitigate issues of gradient vanishing or explosion during the early training phase, thereby improving both training stability and the generalization capability of the model.

In summary, the transformation module significantly enhances the nonlinear processing capability for memoryless Gaussian source sequences through its deep architecture, combined use of activation functions and batch normalization, and appropriate parameter initialization strategies. As a result, it generates more structurally compatible features, which are better adapted to subsequent structured quantization.

### 2.2. Structured Encoding Module

The existing source compression systems typically employ the LDPC–based BP algorithm. However, when directly applied to different kinds of sources, the standard BP algorithm often leads to considerable distortion amplification and high computational complexity. To mitigate these limitations, the proposed system introduces a novel quantization scheme that combines deep neural networks with the BP algorithm, thereby enabling more efficient and robust compression of Gaussian sources.

Specifically, the system first employs a neural network encoder to transform the input continuous Gaussian source sequence into continuous feature representations. To enhance the robustness of quantization, random perturbations are introduced into these features. The perturbed features are subsequently quantified using the BP algorithm to generate discrete output symbols. To facilitate end–to–end training, the quantization module incorporates the STE technique, which approximates gradients through the otherwise non–differentiable quantization operation.

The proposed quantization module integrates neural encoding with BP–based structured quantization within an end–to–end trainable framework. Gaussian source sequences are first encoded into continuous features, perturbed by uniform noise to improve robustness, and then quantized through the BP message passing. A perturbation–enhanced STE is employed to approximate gradients, enabling effective end–to–end network training. This hybrid design effectively combines the structural advantages of the BP with the expressive power of deep neural networks, significantly improving compression performance and system adaptability.

The procedure of the proposed quantization process is outlined in Algorithm 1. The system first applies a neural network encoder E(·) to transform the input Gaussian source sequence x into a continuous feature vector as z=E(x). To enhance the robustness, a uniformly distributed random perturbation u∼U(−0.5,0.5) is added to the continuous feature vector z, resulting in a perturbed feature vector $\tilde{z}=z+u$. Firstly, the symbols of Algorithm 1 are defined as follows:

$\begin{array}{cc} x\in R^{d} & \mathrm{Input}\;\mathrm{Gaussian}\;\mathrm{source}\;\mathrm{sequence}\\ z\in R^{n} & \mathrm{Feature}\;\mathrm{vector}\;\mathrm{from}\;\mathrm{the}\;\mathrm{transformation}\;\mathrm{module}\\ E(\cdot ) & \mathrm{Neural}\;\mathrm{network}\;\mathrm{encoder}\\ Q_{\mathrm{BP}}\left(\cdot \right) & \mathrm{BP}–\mathrm{based}\;\mathrm{quantization}\\ u∼U\left(-0.5,0.5\right),\;u\in R^{n} & \mathrm{Random}\;\mathrm{perturbation}\\ a_{d} & \mathrm{Step}\;\mathrm{size}\;\mathrm{for}\;\mathrm{gradient}\;\mathrm{perturbation}\\ N_{\mathrm{iter}} & \mathrm{Max}\;\mathrm{backpropagation}\;\mathrm{steps}\;\mathrm{during}\;\mathrm{training}\\ BP_{\mathrm{iter}} & \mathrm{Max}\;\mathrm{number}\;\mathrm{of}\;\mathrm{BP}\;\mathrm{iterations}\\ q_{\mathrm{raw}},\;q\in R^{n} & \mathrm{Binary}\;\mathrm{sequence}\;\mathrm{from}\;\mathrm{the}\;\mathrm{BP}\;\mathrm{quantization} \end{array}$


**Algorithm 1** Quantization: BP neural network based on the STE
**Input:** 

$x,E\left(\cdot \right),Q_{\mathrm{BP}}\left(\cdot \right),a_{d},BP_{\mathrm{iter}},N_{\mathrm{iter}}$

**Output:** 

q


 1:**function** BP_Quantization($x,E\left(\cdot \right),Q_{\mathrm{BP}}\left(\cdot \right),a_{d},BP_{\mathrm{iter}},N_{\mathrm{iter}}$) 2:    z←E(x) 3:    u←Uniform(−0.5,0.5) 4:    $\tilde{z}\leftarrow z+u$ 5:    $q_{\mathrm{raw}}\leftarrow Q_{\mathrm{BP}}\left(\tilde{z},BP_{\mathrm{iter}}\right)$ 6:    $q\leftarrow q_{\mathrm{raw}}-u$ 7:    **if** In_Training_Mode **then** 8:        $z_{+}\leftarrow z+a_{d}$ 9:        $z_{-}\leftarrow z-a_{d}$10:        $q_{+}\leftarrow Q_{\mathrm{BP}}\left(z_{+},BP_{\mathrm{iter}}\right)$11:        $q_{-}\leftarrow Q_{\mathrm{BP}}\left(z_{-},BP_{\mathrm{iter}}\right)$12:        $\nabla \leftarrow q_{+}-q_{-}$13:        Update_Encoder_Parameters(∇)14:    **end if**15:    **return** q16:
**end function**




Subsequently, the perturbed feature y is input into the quantization $Q_{\mathrm{BP}}\left(\cdot \right)$, where multi–step BP is performed to obtain an initial discrete quantization result, as

(4) $$q_{raw}=Q_{BP}\left(y\right),$$

where y indicates the continuous feature, and the system outputs the result after perturbation correction as

(5) $$q=q_{raw}-u,$$

where the final output q serves as the result of the current quantization round. The detailed procedure of the BP–based quantization subroutine $Q_{\mathrm{BP}}\left(\cdot \right)$ is presented in Algorithm 2, and the variable definitions are listed as follows:

$\begin{array}{cc} y\in R^{n} & \mathrm{Continuous}\;\mathrm{feature}\;\mathrm{to}\;\mathrm{be}\;\mathrm{quantified}\;(e.g.,\;\tilde{z})\\ BP_{\mathrm{iter}} & \mathrm{Maximum}\;\mathrm{number}\;\mathrm{of}\;\mathrm{BP}\;\mathrm{iterations}\\ H\in {\left\{0,1\right\}}^{m\times n} & \mathrm{Parity}–\mathrm{check}\;\mathrm{matrix}\;\mathrm{of}\;\mathrm{LDPC}\;\mathrm{codes}\\ \mathrm{BP}\_\mathrm{Initialize}(\cdot ) & \mathrm{Initialization}\;\mathrm{function}\;\mathrm{for}\;\mathrm{BP}\;\mathrm{messages}\\ \mathrm{BP}\_\mathrm{Iterate}(\cdot ) & \mathrm{Sin}\mathrm{gle}\;\mathrm{iteration}\;\mathrm{of}\;\mathrm{BP}\;\mathrm{message}\;\mathrm{updating}\\ \mathrm{BP}\_\mathrm{MakeDecision}(\cdot ) & \mathrm{Function}\;\mathrm{for}\;\mathrm{final}\;\mathrm{decision}\;(e.g.,\;\mathrm{sign})\\ q_{\mathrm{raw}}\in {\left\{0,1\right\}}^{n} & \mathrm{Discrete}\;\mathrm{output}\;\mathrm{symbols}\;\mathrm{from}\;\mathrm{BP}\;\mathrm{quantization} \end{array}$

**Algorithm 2** BP quantization subroutine
**Input:** y, $BP_{\mathrm{iter}}$, H**Output:** 

$q_{\mathrm{raw}}$


 1:**function** BP_Quantization($y,BP_{\mathrm{iter}},H$) 2:    Step 1: Initialization 3:    llr←y 4:    BP_state←BP_Initialize(llr,H) 5:    Step 2: Belief Propagation Iterations 6:    **for** 1 to $BP_{\mathrm{iter}}$ **do** 7:        BP_state←BP_Iterate(BP_state,H) 8:    **end for** 9:    Step 3: Final Decision10:    $q_{\mathrm{raw}}\leftarrow \mathrm{BP}\_\mathrm{MakeDecision}\left(BP\_state\right)$11:    **return** $q_{\mathrm{raw}}$12:
**end function**




In Algorithm 2, the initial BP internal message state is denoted as ${BP_{state}}_{0}$. In each round of BP iteration, the message updates between check nodes c and variable nodes v follow the rules defined in Algorithm 3. In this way, the training loop of the proposed system is completed. The message passing between check nodes and variable nodes is formulated by the following equations:

(6) $$m_{c\to v}=2\tanh^{-1}\left(\prod_{v^{\prime }\in V\left(c\right)\setminus v}\tanh\left(\frac{m_{v^{\prime }\to c}}{2}\right)\right),$$

(7) $$m_{v\to c}=llr_{v}+\sum_{c^{\prime }\in C\left(v\right)\setminus c}m_{c^{\prime }\to v^{\prime }},$$

where $m_{c\to v}$ and $m_{v\to c}$ are the check–to–variable and variable–to–check messages, respectively. The symbol $v^{\prime }$ denotes another variable node that is connected to check node *c*, and $v^{\prime }\in V\left(c\right)\setminus v$ indicates the set of all variable nodes connected to *c* except *v*. Similarly, $c^{\prime }\in C\left(v\right)\setminus c$ represents the set of all check nodes connected to *v* except *c*. The term $m_{c^{\prime }\to v^{\prime }}$ represents the message sent from check node $c^{\prime }$ to variable node $v^{\prime }$ in the previous iteration.

After several iterations of the BP, the marginal probability estimation of variable node is given by

(8) $$LLR_{v}=llr_{v}+\sum_{c\in C(v)}m_{c\to v}.$$

A final decision is then applied to obtain the quantized output, as

(9) $$q_{raw,v}=\mathrm{sign}\left(LLR_{v}\right),$$

where sign(·) represents the symbolic function. All variable node decisions are aggregated to form $q_{raw}$, which serves as the output of the current BP–based quantization process.

In Algorithm 3, the message updating rules between check nodes c and variable nodes v are presented. The variables are defined as follows:

$\begin{array}{cc} q_{\mathrm{raw}}\in R^{n} & \mathrm{Quantized}\;\mathrm{input}\;\mathrm{to}\;\mathrm{decoder}\;(\mathrm{from}\;\mathrm{BP}\;\mathrm{quantization})\\ s^{\prime }\in R^{d} & \mathrm{Reconstructed}\;\mathrm{signal}\;\mathrm{from}\;\mathrm{decoder}\\ W_{i},\;b_{i} & \mathrm{Weight}\;\mathrm{and}\;\mathrm{bias}\;\mathrm{of}\;\mathrm{layer}\;i\\ h_{i} & \mathrm{Output}\;\mathrm{of}\;\mathrm{layer}\;i\;\mathrm{before}\;\mathrm{activation}\\ \mathrm{BatchNorm}(x) & \mathrm{Batch}\;\mathrm{normalization}\;\mathrm{layer}\\ \mathrm{LeakyReLU}(x) & \mathrm{Activation}\;\mathrm{function}\;(\alpha =0.01)\\ \mathrm{Tanh}(x) & \mathrm{Hyperbolic}\;\tan\mathrm{gent}\;\mathrm{activation}\\ \mathrm{Dropout}(x,p) & \mathrm{Randomly}\;\mathrm{drops}\;p=0.3\;\mathrm{of}\;x\\ \mathrm{Init}. & \mathrm{Xavier}\;\mathrm{for}\;W_{i},\;\mathrm{zeros}\;\mathrm{for}\;b_{i} \end{array}$

**Algorithm 3** Postprocessing of quantization
**Input:** 

$q_{\mathrm{raw}}\in R^{n}$

**Output:** 

$s^{\prime }\in R^{d}$


 1:Layer 1: Linear + BN + LeakyReLU 2:

$h_{1}\leftarrow q_{\mathrm{raw}}W_{1}+b_{1}$

 3:

$h_{1,\mathrm{BN}}\leftarrow \mathrm{BatchNorm}\left(h_{1}\right)$

 4:

$h_{1,\mathrm{ACT}}\leftarrow \mathrm{LeakyReLU}\left(h_{1,\mathrm{BN}}\right)$

 5:Layer 2: Linear + BN + LeakyReLU 6:

$h_{2}\leftarrow h_{1,\mathrm{ACT}}W_{2}+b_{2}$

 7:

$h_{2,\mathrm{BN}}\leftarrow \mathrm{BatchNorm}\left(h_{2}\right)$

 8:

$h_{2,\mathrm{ACT}}\leftarrow \mathrm{LeakyReLU}\left(h_{2,\mathrm{BN}}\right)$

 9:Layer 3: Linear + BN + LeakyReLU10:

$h_{3}\leftarrow h_{2,\mathrm{ACT}}W_{3}+b_{3}$

11:

$h_{3,\mathrm{BN}}\leftarrow \mathrm{BatchNorm}\left(h_{3}\right)$

12:

$h_{3,\mathrm{ACT}}\leftarrow \mathrm{LeakyReLU}\left(h_{3,\mathrm{BN}}\right)$

13:Layer 4: Linear + Tanh14:

$h_{4}\leftarrow h_{3,\mathrm{ACT}}W_{4}+b_{4}$

15:

$s_{\mathrm{raw}}^{\prime }\leftarrow \mathrm{Tanh}\left(h_{4}\right)$

16:Dropout (applied during training)17:

$s^{\prime }\leftarrow \mathrm{Dropout}\left(s_{\mathrm{raw}}^{\prime },p=0.3\right)$

18:**return** $s^{\prime }$



During neural network training, to address the non–differentiability of the quantization operation, the following STE is adopted to approximate the gradient of the quantization function as

(10) $$\frac{\partial q}{\partial z}\approx Q_{BP}\left(z+a_{d}\right)-Q_{BP}\left(z-a_{d}\right),$$

where $a_{d}$ denotes the gradient perturbation amplitude, which is typically set to $a_{d}=\sqrt{\frac{1}{2}}$. The symmetric–difference step is set to $a_{d}=\sqrt{\frac{1}{2}}$, which balances the gradient signal–to–noise ratio under the added uniform noise and prevents saturation in the STE. The balance performance is observed in $a_{d}\in \left[0.5,0.8\right]$, and the default $a_{d}=\sqrt{\frac{1}{2}}$ works robustly across all settings.

### 2.3. Decoding Module

**Figure 3:**
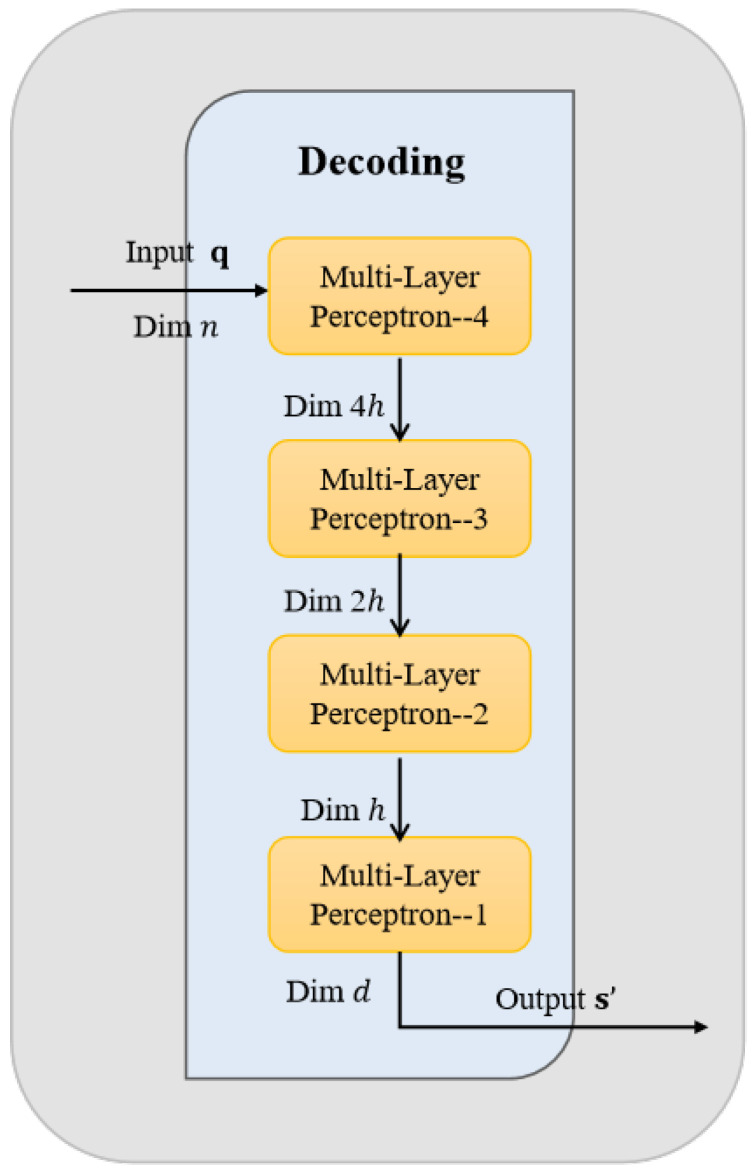
Detailed architecture of the proposed decoder.

The decoding module reconstructs an approximate version of the original Gaussian source signal from the binary codeword sequence output by the encoder–side quantization module, as illustrated in Figure 3. Given a codeword sequence of length n, denoted as $q=\{q_{1},q_{2},\ldots ,q_{n}\}$, the reconstruction module first maps it back into the continuous domain to produce a signal $s^{\prime }=\left\{s_{1}^{\prime },s_{2}^{\prime },\ldots ,s_{n}^{\prime }\right\}$, which has the same dimensionality as the original input. The internal structure of the MLP in the decoding module is shown in Table 3. Here, *n* denotes the length of the quantified binary codeword, *d* represents the dimension of the reconstructed signal, and the variables satisfy $q_{i}\in \left\{0,1\right\}$ and $s_{i}^{\prime }\in R$.

The output dropout indicates that the dropout =0.3 is applied only before the last hour during training, and it is disabled during reasoning. The input codeword q is produced by the quantization module and possesses the same dimensionality as the original input signal. Although the quantization is structurally designed based on the P–LDPC code, yielding a high compression rate, it inherently lacks continuous expressiveness. To overcome this limitation, the reconstruction module performs a nonlinear inverse mapping that converts the binary codeword back into a continuous–valued signal.

Common activation functions present distinct trade–offs: ReLU–family activations are non–saturating and promote sparsity but risk dead neurons; GELU provides smooth, stochastic gating at a higher computational cost; and Tanh, while saturating, naturally bounds outputs to [−1,1]. Based on these properties, we employ LeakyReLU (α=0.01) in hidden layers to mitigate the dead neuron issue and ensure stable gradient flow, and tanh at the output layer to constrain the pre–quantization signal. This architecture was empirically found to offer the best trade–off between convergence stability and rate–distortion performance.

As shown in Figure 3, the MLP is symmetrically implemented in the transformation module. The reconstruction comprises a series of fully connected layers. Each linear layer is followed by batch normalization and a LeakyReLU activation function. The output layer uses a tanh activation to constrain the amplitude of the reconstructed signal within the range (−1,1). Hence, the numerical instability is prevented, and the output is aligned with the distribution of the original Gaussian source.

Assuming a batch size of *p* and input codewords $q\in R^{p\times n}$, the reconstruction process is expressed as the following function:

(11) $$s^{\prime }=\tanh\left(g_{L}\left(\cdots g_{2}\left(g_{1}\left(q\right)\right)\right)\right),$$

where the mapping function at the *i*-th layer is denoted as $g_{i}\left(\cdot \right)$ as

(12) $$g_{i}\left(x\right)=\mathrm{LeakyReLU}(\mathrm{BatchNorm}\left(xW_{i}+b_{i}\right)),$$

where $W_{i}$ and $b_{i}$ indicate the weight matrix and bias vector of the *i*-th layer, respectively.

By incorporating batch normalization layers, the model dynamically normalizes the mean and variance of each layer’s outputs during training, which accelerates convergence and enhances training stability. The LeakyReLU activation function preserves the sparsity properties of ReLU while alleviating the dying neuron issue, thereby strengthening the nonlinear representational capacity. The output layer employs a tanh activation function to confine the reconstructed signal within the appropriate numerical range, consistent with the statistical properties of the Gaussian source. Hence, the reconstruction quality is improved.

The entire neural network is trained by minimizing the mean squared error (MSE) between the original Gaussian source s and the reconstructed signal $s^{\prime }$, which is formulated as follows:

(13) $$L_{\mathrm{MSE}}=\frac{1}{pn}\sum_{i=1}^{p}\sum_{j=1}^{n}{\left(s_{ij}-s_{ij}^{\prime }\right)}^{2},$$

where $L_{\mathrm{MSE}}$ denotes the mean squared error, *p* represents the number of samples processed in parallel (i.e., batch size), *n* is the dimensionality of each sample, corresponding to the length of the Gaussian source sequence, $s_{ij}$ is the *j*-th element of the *i*-th original sample, and $s_{ij}^{\prime }$ is the corresponding reconstructed value produced by the decoding module. The term ${\left(s_{ij}-s_{ij}^{\prime }\right)}^{2}$ represents the squared reconstruction error for the *j*-th component of the *i*-th sample. The double summation $\sum_{i=1}^{p}\sum_{j=1}^{n}$ aggregates the squared error over all elements of all samples. Finally, the normalization factor $\frac{1}{pn}$ computes the average error over the entire batch.

The MSE measures quantifies the pointwise average difference between the network output $s^{\prime }$ and the ground–truth signal s, where the lower MSE indicates better reconstruction capability and higher compression fidelity. In contrast to traditional decoders that rely on lookup tables or shallow neural networks, the reconstruction module proposed in this work demonstrates significantly enhanced feature restoration and information recovery performance. Without requiring access to the original signal or intermediate continuous features from the encoder, the decoder reconstructs the signal directly from a single–path binary codeword. This approach substantially simplifies the decoding process, improves decoding efficiency, and enhances the practicality of the end–to–end compression system.

## 3. System Optimization and Technical Details

### 3.1. Gradient Backpropagation

In this section, the non–differentiability issue is investigated in the joint training of the BP–based quantizer and the neural network. Since the quantization module implemented through the BP algorithm is embedded as a layer within the neural network, it should supply informative gradients during backpropagation. These gradients are necessary to update the parameters of preceding network layers and to minimize the overall loss function. However, the quantization function is inherently discontinuous and non–differentiable. Direct gradient computation results in values that are zero almost everywhere. This leads to gradient truncation in the backward pass, which prevents effective parameter updates and hinders end–to–end optimization.

To address the aforementioned challenge, the STE strategy is employed to approximate the gradients of the quantization function, thereby facilitating effective gradient backpropagation throughout the network. Specifically, random perturbation is introduced to the continuous feature vector before quantization. The resulting perturbed outputs are then used to approximate the derivative of the otherwise non–differentiable function through a finite–difference method. This approximation process, detailed in Algorithm 3, adheres to the principles of the STE and enables stable gradient propagation across the non–differentiable quantization layer during training.

Let z be a continuous input feature vector. By introducing a uniformly distributed random perturbation u∼U(−0.5,0.5), the perturbed feature vector is obtained as $\tilde{z}=z+u$. Then, the derivative of the quantization function Q(·) in the expectation sense is expressed as

(14) $$\begin{array}{cc} \frac{d}{dz}E\left[Q\left(z+u\right)\right] & =\frac{d}{dz}\int_{-t/2}^{t/2}Q\left(z+v\right)dv\\ \; & =Q(z+t/2)-Q(z-t/2), \end{array}$$

where Q(·) denotes the quantization function based on the BP algorithm, and E[·] represents the expectation operator. The quantization step size and the perturbation range follow a uniform distribution with U(−t/2,t/2). The difference form in the above expression provides a numerically meaningful approximation of the gradient for the non–differentiable function, thereby enabling the propagation of informative gradient signals and supporting effective training of the neural network.

The STE configuration employs a uniform perturbation, u∼U(−0.5,0.5), and a symmetric–difference step size of $a_{d}=\sqrt{1/2}$. The underlying neural network utilized the LeakyReLU activation function with a negative slope of 0.01 and was initialized using the Xavier–uniform method, with biases set to zero. The LeakyReLU with a negative slope α=0.01 is used in hidden layers. All biases are zero–initialized, while the final tanh layer uses Xavier uniform. These settings stabilize optimization and are compatible with the STE defined in Equations (14) and (15). In the implementation, the system adopts a fixed perturbation step size. The approximate gradient of the quantization function is then computed using the symmetric difference form as

(15) $$\frac{\partial Q(z)}{\partial z}\approx Q\left(z+a_{d}\right)-Q\left(z-a_{d}\right),$$

where the size of the fixed perturbation step is given as $a_{d}=\sqrt{\frac{1}{2}}$.

The proposed gradient approximation facilitates stable, end–to–end quantization training by effectively mitigating the vanishing gradient problem. This method seamlessly integrates the quantization into the learning process, enabling accurate gradient propagation and bridging the train–inference gap while preserving standard BP. Consequently, it substantially improves both training stability and final quantization accuracy.

Unless otherwise specified, models are trained for 4000 iterations with the Adam optimizer (learning rate of $1\times 10^{-4}$, $\beta_{1}=0.9$, $\beta_{2}=0.999$), a batch size of 1000, and gradient clipping at ${\left|\nabla \right|}_{2}\leq 1.0$. The STE employs a uniform perturbation u∼U(−0.5,0.5), a symmetric–difference step $a_{d}=\sqrt{1/2}$, LeakyReLU activations (α=0.01), and Xavier/He initialization with zero biases. Evaluation is performed on a fixed set of 10,000 i.i.d. Gaussian samples, with results reported as the mean and ±1 standard deviation over five independent runs.

The proposed framework is designed as an end–to–end trainable network, with the original continuous input sequence serving as the training target. The loss function is defined as the MSE between the input and the reconstructed signal. Optimization is carried out using the Adam algorithm with a learning rate of 0.0001 and a batch size of 1000. The model undergoes 4000 training iterations to ensure stable convergence. A detailed summary of the training configuration is provided in Table 4.

### 3.2. Complexity Analysis

The overall training complexity of the proposed end–to–end compression framework is decomposed into three major components, including the encoder network, the structured quantization module, and the decoder network.

In the encoder module, the encoder consists of $L_{\mathrm{enc}}$ fully connected layers, each with a maximum hidden width $h_{\max,\mathrm{enc}}$. For a batch size of *B*, the time complexity of the encoder per training step is $O(B\cdot L_{\mathrm{enc}}\cdot h_{\max,\mathrm{enc}}^{2})$ and the corresponding space complexity is $O(L_{\mathrm{enc}}\cdot h_{\max,\mathrm{enc}}^{2})$.

In the quantization module, there are subcomponents containing the additive noise injection and the BP algorithm. The additive perturbation step introduces uniform noise into each *n*-dimensional encoder output, resulting in a time complexity of O(B·n). The BP process operates on a P–LDPC matrix containing *E* non–zero entries and executes *I* iterations per decoding, leading to a time complexity of O(B·E·I).

In the decoder module, the structure mirrors that of the encoder, consisting of $L_{\mathrm{dec}}$ fully connected layers with a maximum width of $h_{\max,\mathrm{dec}}$. The time and space complexities per forward pass are $O(B\cdot L_{\mathrm{dec}}\cdot h_{\max,\mathrm{dec}}^{2})$ and $O(L_{\mathrm{dec}}\cdot h_{\max,\mathrm{dec}}^{2})$, respectively.

The total computational complexity integrates all components. The overall time complexity per training iteration is $O\left(B\cdot \left(L_{\mathrm{enc}}\cdot h_{\max,\mathrm{enc}}^{2}+E\cdot I+L_{\mathrm{dec}}\cdot h_{\max,\mathrm{dec}}^{2}\right)\right)$, and the space complexity is $O\left(L_{\mathrm{enc}}\cdot h_{\max,\mathrm{enc}}^{2}+L_{\mathrm{dec}}\cdot h_{\max,\mathrm{dec}}^{2}\right)$.

This analysis demonstrates that although the structured quantization module incurs additional computational cost through the BP, the process remains computationally tractable owing to the sparse structure of the LDPC matrix. Furthermore, the computational demands of the encoder and decoder modules scale quadratically with network width yet linearly with batch size and network depth, thereby facilitating efficient training on GPU architectures.

## 4. Simulation Results and Analyses

To evaluate the effectiveness of the proposed end–to–end neural network integrated with P–LDPC codes for lossy compression, a comprehensive set of comparative experiments is conducted. All experiments are performed under identical datasets and training configurations to ensure fairness. Training loss curves are recorded throughout the optimization process to assess the performance and generalization capability.

In this section, a standard Gaussian source X∼N(0,1) is used as a representative example. The MSE is used as the distortion measure, and the theoretical lower bound of the distortion–rate function is given by [29]

(16) $$D\left(R\right)=\sigma^{2}\cdot 2^{-2R},$$

where D is the distortion, *R* is the compression rate in bits per dimension, and $\sigma^{2}=1$ for the standard Gaussian source.

The parity–check matrix of the proposed P–LDPC codes is constructed and extended using the progressive edge growth algorithm [30], designed to maximize the girth of the factor graph while preserving sparsity, thereby enhancing decoding performance. In the implemented system, the compression rate *R* is determined by the encoding matrix $H\in F_{2}^{(1-r)n\times n}$ and is defined as

(17) $$R=\frac{n-m}{n},$$

where *n* is the codeword length, *m* is the number of parity–check equations, and H is a sparse parity–check matrix of dimension m×n.

A higher compression rate *R* corresponds to reduced redundancy, thereby improving compression efficiency at the potential cost of increased decoding difficulty under low–distortion constraints. To evaluate the system across varying compression strengths, the row–to–column ratio of the PEG–generated matrix H is adjusted, enabling flexible control over the rate *R*.

**Figure 4:**
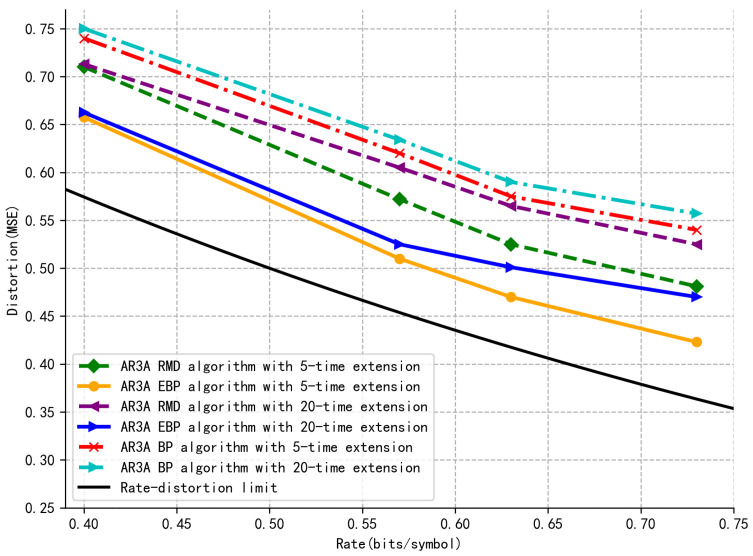
Distortion–rate based on the AR3A code with varying extension times and decoders.

The extension factor *k* determines the scale of the protograph, defining a parity–check matrix of size $\left(km_{0}\right)\times \left(kn_{0}\right)$. Scaling up *k* increases computational complexity due to growth in both the number of edges *E* and the BP iterations *I*. Therefore, reducing *k* lowers the system runtime. This reduction also tends to yield lower distortion for a fixed–capacity MLP, as it operates on a lower–dimensional target, aligning with the trends observed in Figure 4. This scaling increases the block length *n* and the number of check nodes *m*, thereby influencing both the encoding complexity and the resulting distortion.

In Figure 4, the distortion–rate performance based on the benchmark P–LDPC code AR3A [31] is compared with the RMD–based system [21], the conventional BP–based system [32], and the proposed EBP–based system. The benchmark code is extended to be 5 and 20 times, respectively. It is clear that the proposed system has lower distortion over 0.05 at the same rate. In addition, fewer extensions of the P–LDPC code present less distortion performance, which indicates that a lower dimension will decrease the output distortion and coding runtime simultaneously. In this case, the complexity of the proposed EBP–based system will be effectively reduced.

**Figure 5:**
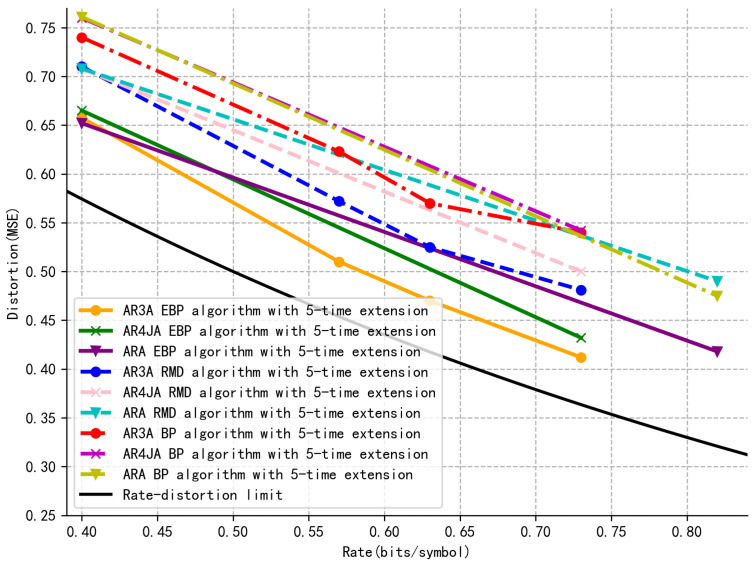
Distortion–rate performance based on the AR3A, AR4JA, and ARA codes.

In Figure 5, the distortion–rate performance of the BP, RMD, and EBP algorithms is compared. The simulations are conducted using three benchmark P–LDPC codes as proposed in [31], including AR3A, ARA, and AR4JA. It is evident that the AR3A code achieves less distortion, approaching the theoretical distortion–rate bound more closely. Among the different methods, the EBP algorithm using the AR3A code demonstrates better performance than the conventional, indicating that the EBP is more efficient than both the RMD and the BP in terms of rate–distortion performance.

The parameters *n* and *m* for the evaluated codes are given as follows. The codes ARA0.4 and AR4JA0.4 both correspond to n=25 and m=15, while the codes ARA0.82 and AR4JA0.73 correspond to n=85, m=15 and n=55, m=15, respectively. These configurations demonstrate the variation in codeword length *n* and number of parity–check equations *m* across different code rates and structures.

**Figure 6:**
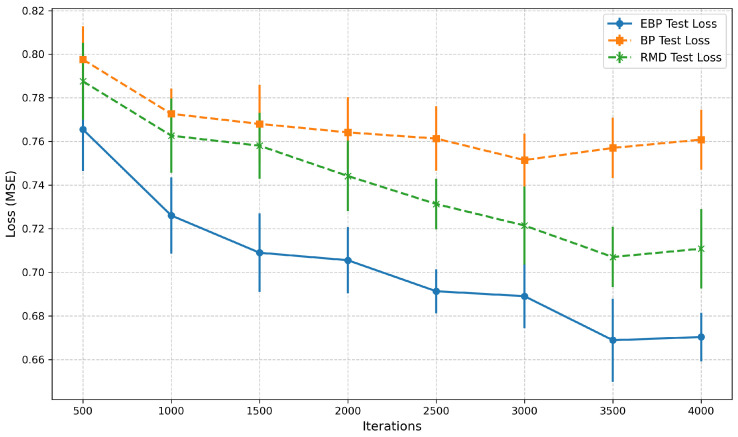
Loss–iterations comparisons among three methods.

As illustrated in Figure 6, the proposed EBP algorithm exhibits superior convergence and lower MSE loss compared to both conventional BP and the RMD algorithm during testing. The EBP reaches a stable test loss of approximately 0.69 after 4000 iterations, demonstrating a smoother convergence trajectory. In contrast, the conventional BP and RMD stabilize around 0.76 and 0.73, respectively.

Furthermore, the curves represent the mean over five independent runs, with error bars indicating the standard deviation. An obvious improvement of nearly 0.12 for EBP over the conventional methods. The proposed EBP algorithm shows markedly smaller error bars than the benchmarks, signifying greater robustness to random initialization. While the BP exhibits significant variability, especially initially, the RMD algorithm demonstrates intermediate stability. These results confirm that the proposed EBP method achieves superior performance, offering faster convergence, a lower final test loss, and enhanced stability compared to the BP and RMD algorithms, as evidenced by its smaller error bars and smoother convergence trajectory.

Overall, the proposed system substantially improves the quantization accuracy and reconstruction performance by integrating deep neural transformations, random perturbations, and the STE strategy. Even on variable data, it consistently achieves lower reconstruction distortion, demonstrating that the proposed method exhibits not only enhanced fitting capability but also significantly stronger generalization in end–to–end lossy compression of Gaussian sources. These attributes contribute to more reliable performance in practical deployment scenarios.

In a representative setting (code length n=100, batch size B=1), the inference latency was measured on a single GPU, as shown in Table 5. While the core BP kernel per iteration is identical for both BP and EBP, the proposed EBP incurs only a negligible overhead from an additional encoder/decoder forward pass (approximately 0.10 ms per sample). This results in a total latency that is only 1.03 times that of standard BP at I=10 iterations.

## 5. Conclusions

This paper introduces an end–to–end lossy source coding system combining the multi–layer perceptron neural network. By integrating deep nonlinear transformations, stochastic perturbations, and a straight–through estimator, the proposed approach effectively resolves the non–differentiability issue in the source quantization. Simulations show that the proposed system surpasses conventional methods in distortion–rate performance and generalization, while also converging faster and achieving lower distortion compared to classical P–LDPC codes. The framework maintains manageable complexity through reduced matrix dimensions and decoding iterations, offering an efficient and practical solution for the source compression in sensor networks.

## Figures and Tables

**Table 1:** Notations used throughout the paper.

Symbol	Meaning
*n*	Codeword length (bits)
*m*	Number of parity–check equations
R=(n−m)/n	Compression rate (bits/dim)
*d*	Signal dimension (input/output)
$q\in {\left\{0,1\right\}}^{n}$	Quantized binary codeword
$z\in R^{n}$	Encoder feature vector
u∼U(−0.5,0.5)	Uniform perturbation for robustness/STE
$a_{d}$	Symmetric–difference step in STE (default $a_{d}=\sqrt{1/2}$)
E,I	Nonzeros in parity–check matrix of BP iterations
$L_{\mathrm{enc}/\mathrm{dec}},\;h_{\max,\mathrm{enc}/\mathrm{dec}}$	Layers and the maximum hidden width of the encoder or decoder

**Table 2:** Internal structure of the MLP in the encoding module.

Layer	Type	Input Dim.	Output Dim.	Activation
Input	–	*d*	*d*	–
Layer 1	Linear + BN	*d*	2(d+n)	LeakyReLU
Layer 2	Linear + BN	2(d+n)	4(d+n)	LeakyReLU
Layer 3	Linear + BN	4(d+n)	8(d+n)	LeakyReLU
Layer 4	Linear	8(d+n)	*n*	Tanh
Output	–	*n*	*n*	–

**Table 3:** Internal structure of the MLP in the decoding module.

Layer	Type	Input Dim.	Output Dim.	Activation
Input	-	*n*	*n*	-
Layer 1	Linear + BN	*n*	8(d+n)	LeakyReLU
Layer 2	Linear + BN	8(d+n)	4(d+n)	LeakyReLU
Layer 3	Linear + BN	4(d+n)	2(d+n)	LeakyReLU
Layer 4	Linear	2(d+n)	*d*	Tanh
Output	Dropout	-	*d*	-

**Table 4:** Training hyperparameters of the proposed network.

Hyperparameter	Training
Loss Function	MSE
Optimizer	Adam
Learning Rate	0.0001
Batch Size	1000
Maximum Iterations	4000
Gradient Approximation	STE

**Table 5:** The inference latency comparisons.

Method	Iterations *I*	BP Time/Iter	MLP Fwd.	Total/Sample
BP–only	10	0.35 ms	–	3.50 ms
EBP	10	0.35 ms	0.10 ms	3.60 ms
Relative ratio (EBP/BP): 1.03×				

## Data Availability

Data is contained within the article.
